# Personal

**Published:** 1915-01

**Authors:** 


### PERSONAL.

---

Announcement of removal, travel, and other matters of interest are requested. Please report errors in the listing of any physician in the State and other directories, that we may co-operate with the State Society in securing a correct list.

---

Dr. O. M. Grover of Dunkirk has been appointed to the associate staff of the Bedford Hills State Reformatory for Women.

Dr. J. E. Clark is Sanitary Superintendent of Oneida and Herkimer Counties.

---

Leuman M. Waugh, D. D. S., formerly of Buffalo, announces the continuance of his practice at 576 Fifth Avenue, New York.

---

Dr. Malcolm S. Woodbury of Clifton Springs has been appointed Superintendent of Clifton Springs Sanitarium, to succeed the late Dr. James G. Mumford.

---

Dr. F. W. E. Wilson of Niagara Falls, Ont., has joined a cavalry contingent as surgeon.

---

Dr. R. C. Mehnert of Buffalo was attacked by a dog and badly bitten as he was leaving the house of a patient November 26.

---

Dr. George McK. Hall of Buffalo has been unanimously chosen President of the Buffalo Aerie No. 46 of the Eagles.

---

We regret to learn that Dr. John Hutchens of Canandaigua has been in bad health for some months.

---

Dr. Regina Flood Keyes of Buffalo returned from France about the middle of December.

---

Dr. Leslie Miller of Niagara Falls has recently taken a short post graduate course in surgery in New York.

---

Dr. Wm. F. Beck of Buffalo has been elected President of the East Clinton Street Business Men's and Taxpayers' Association.

---

The automobile of Dr. Arthur Eisbein was damaged to the amount of $1,000 by fire from back-firing December 19. It was insured.

